# A new species of *Lesticus* Dejean, 1828 (Coleoptera, Carabidae) from the Finisterre Range, Papua New Guinea and a key to the genera of pterostichine-like Harpalinae of New Guinea

**DOI:** 10.3897/zookeys.246.4112

**Published:** 2012-11-29

**Authors:** Kipling Will, David H. Kavanaugh

**Affiliations:** 1 Essig Museum of Entomology, University of California, Berkeley, CA 94720; 2 Department of Entomology, California Academy of Sciences, San Francisco, CA 94118

**Keywords:** New Guinea, Carabidae

## Abstract

*Lesticus finisterrae* (Carabidae: Pterostichini) **sp. n.** (type locality: Finisterre Range, Papua New Guinea), is described and characters to differentiate it from other “Trigonotomi” species are given. A key to the genera of pterostichine-like Harpalinae of the island, including all genera of Morionini, Cratocerini, Drimostomatini, Abacetini, Loxandrini and Pterostichini, is provided. The genus *Rhytisternus* (Pterostichini) is for the first time reported from New Guinea, represented by the likely adventive species *Rhytisternus laevis* (Macleay). The previously unknown male of *Stegazopteryx ivimkaensis* Will (Drimostomatini) is described.

## Introduction

Darlington reported 667 species of Carabidea from New Guinea in his treatment of the fauna (1971) and a search of the Zoological Record for new species and new records suggests that the total is now easily greater than 700 species. Given the complexity of island’s geological history (Baldwin et al. 2012) and the large areas that remain unsampled for beetles, the true diversity is probably at least double what is now known and some have estimated it could be as high as 2100 species (http://www.papua-insects.nl/insect%20orders/Coleoptera/Carabidae/Carabidae.htm)

**Figure 1.**
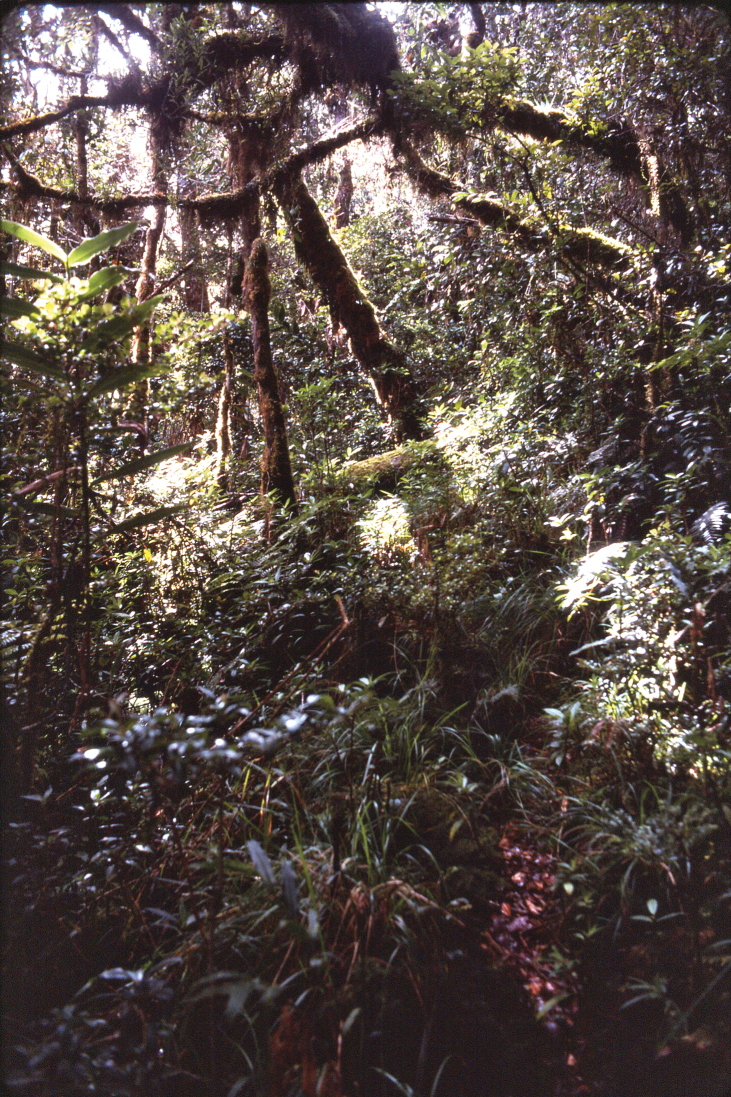
View within upper montane moss forest habitat at 3050m elevation, Finisterre Range, Papua New Guinea. The pitfall trap in which the unique holotype of *Lesticus finisterrae* sp. n. was collected was located in the shaded area just below the middle of the figure.

**Figure 2.**
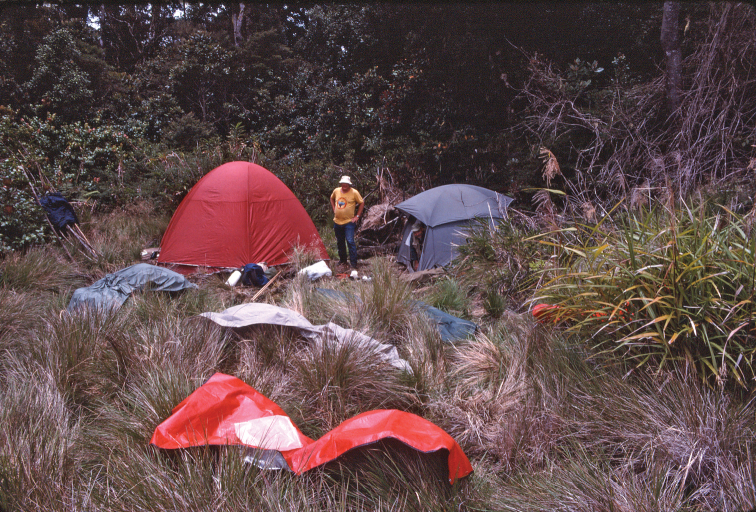
Grassland/upper montane moss forest ecotone at 3050m elevation in the Finisterre Range, Papua New Guinea. The holotype of *Lesticus finisterrae* sp. n. was collected in a pitfall trap placed approximately 20m inside the forest edge.

One area poorly covered by Darlington’s study was the Finisterre Range along the northeastern coast of Papua New Guinea, extending approximately from Madang in the north to Lae in the south. This area was previously recognized as a unique area of endemism (Liebherr 2008). During March and April of 1989, one of us [DHK] had the opportunity to visit this remote and physiographically isolated area and briefly sample the carabid beetle fauna near the highest part of the Finisterre Range, west of the village of Teptep. A challenging three-day uphill trek from Teptep brought our party, which also included George E. Ball of the University of Alberta, Edmonton, and Norman D. Penny of the California Academy of Sciences, to some of the highest forest in the region and to the ecotone between this upper montane moss forest and the grassland that replaces it at higher elevations (Fig. 1–2).

The forest canopy was between 10 and 20 meters tall, dense, with a lush coating of mosses and other epiphytes on tree trunks, branches, logs and stones on the forest floor. A low and fairly dense understory obscured most of the forest floor and made collecting difficult. Consequently, a few pitfall traps were installed in this cool and relatively dark habitat (Fig. 1). After two nights in the ground, the traps were collected and their contents examined. Only a single carabid beetle was found in the catch and the specimen was distinctly different from anything that has been described. Subsequent comparative microscopic study and dissection have confirmed this conclusion.

The genus *Lesticus* Dejean in New Guinea was treated by Darlington (1962, 1971) and again more recently by Dubault et al. (2008). The informal name **“**Trigonotomi” sensu Dubault et al. (2008) is used here as a working hypothesis for a presumably related group of species that includes members of *Lesticus*, *Trigonotoma* Dejean, *Euryaptus* Bates, *Pareuryaptus* Dubault, Lassalle & Roux and *Nesites* Andrewes. This complex of genera includes species that extend its range from northern Australia to Asia. Herein we describe this new species, provide a key to all of the pterostichine-like Harpalinae genera that have typically been associated with Pterostichini by Darlington and past authors that followed him. There is no evidence to support the monophyly of a group including all the taxa represented in the key, but their superficial similarity makes it convenient to treat them together for the purpose of identification until a satisfactory revision of the classification of Harpalinae is reached. Additionally, we add a new record for *Rhytisternus* Chaudoir and information on the male of *Stegazopteryx* Will.

## Methods

Methods and terms follow Will (2011).

### Specimens

Comparative material and specimens studied to develop the key are deposited in the following institutional collections: Australian National Insect Collection (ANIC), Canberra, ACT; California Academy of Science (CASC), San Francisco, CA; Essig Museum of Entomology (EMEC), Berkeley, CA; Institute Royal des Sciences Naturelles de Belgique, (IRSNB), Bruxelles; Museum of Comparative Zoology (MCZ), Harvard University, Cambridge, MA; The Natural History Museum, London (NHM); Nationaal Natuurhistorische Museum (NNM), Leiden, Netherlands; Muséum National d’Histoire Naturelle (MNHN), Paris; Queensland Museum (QM), Brisbane; Bohart Museum of Entomology (UCDC), Davis, CA; and Zoologische Staatssammlung (ZMS), München.

## Taxonomy

### Lesticus finisterrae

Will and Kavanaugh sp. n.

urn:lsid:zoobank.org:act:52A9C3D7-A304-4D05-83DC-D1E385182B6F

http://species-id.net/wiki/Lesticus_finisterrae

#### Holotype.

Male. “PAPUA NEW GUINEA, Madang/Morobe Province border, Finisterre Range, Teptep area, 3.5 air km WNW of Kewieng No.4 village, 3050 m, 28 March 1989 stop #89-50// D. H. Kavanaugh, G. E. Ball & N. D. Penny collectors, Cal. Acad. Sci. Specimen//Papua New Guinea Expedition – 1989// California Academy of Sciences Type No. 18684//HOLOTYPE *Lesticus finisterrae* Will & Kavanaugh [red label]”. Deposited CASC.

#### Type locality.

Finisterre Range, Papua New Guinea. 5.99778°S, 52280°W.

#### Diagnosis.

This species shares with other *Lesticus* species sharply hooked mandibular apices, relatively broad mentum, extremely wide gula (nearly the width of the mentum) and has the general form of a modified species of the *Lesticus chloronotus* group. It is distinguished from all other species of *Lesticus*, including the New Guinea species covered in Darlington’s keys and descriptions (1962, 1971) and Dubault et al.’s key (2008), by the combination of three or more setae in elytral intervals 3 and 5, the absence of transverse sulci of abdominal ventrites 4–5 and the presence of extremely prominent eyes.

#### Description.

**Figure 3.**
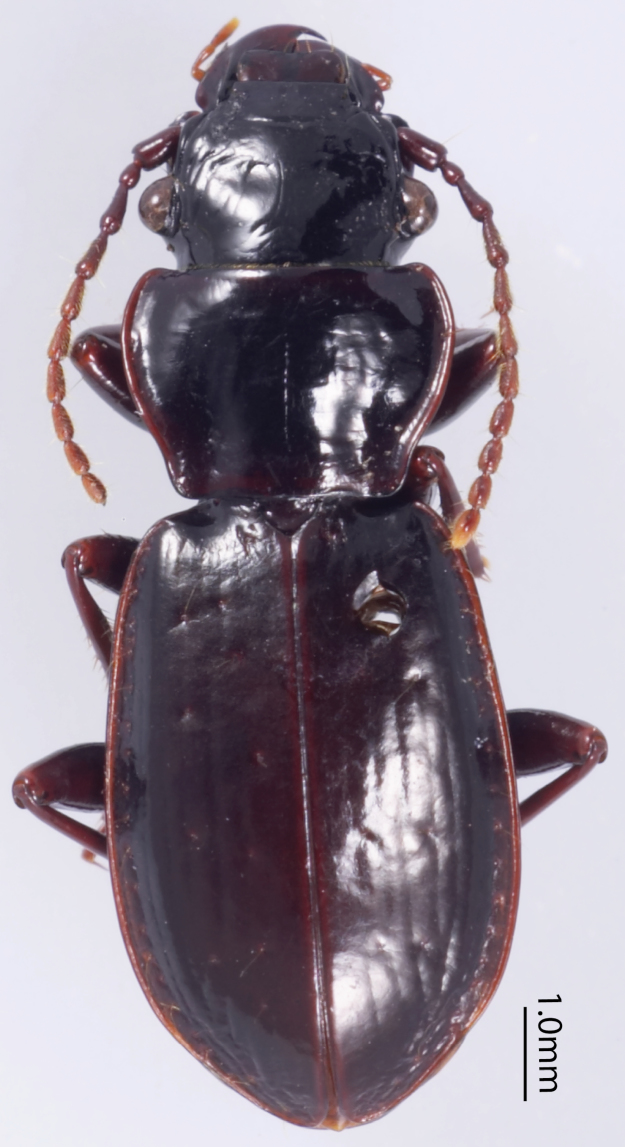
Dorsal habitus of Holotype of *Lesticus finisterrae*.

**Figure 4.**
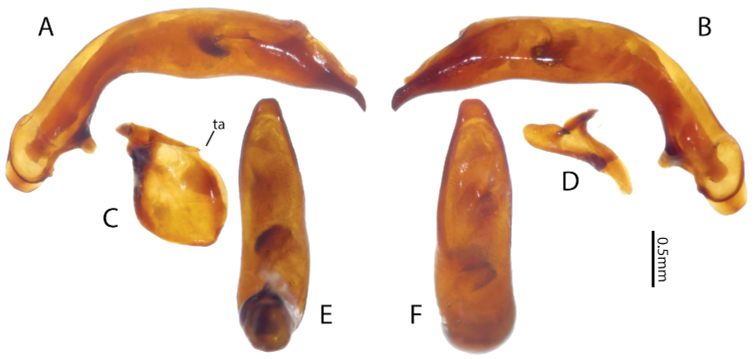
Male genitalia of holotype of *Lesticus finisterrae*. Median lobe. **A** left lateral **B** right lateral **E** ventral and **F** dorsal views. Parameres. **C** left **D** right. **ta** transverse apophysis.

(Fig. 3), *Size*. Overall length (sbl) 20.0mm, greatest width over elytra 8.5mm. *Color*. Dorsal and ventral surfaces black to brunneous. Legs, mouthparts, and antennae slightly paler, lateral margins of pronotum and lateral and sutural areas of elytra margins piceous to rufopiceous. *Luster*. Dorsally and ventrally shiny. *Iridescence*. Elytra and ventral surface of body without spectral iridescence. *Head*. Dorsal microsculpture absent, entire surface with micropunctulae, clypeal-ocular sulci impression absent, shallowly rugose above eyes, with broad, shallow paramedial depressions, ocular ratio (greatest width over eyes/width between eyes at level of anterior supraorbital setae) 1.29, eyes moderately large size, very prominently “bulging”. Labrum with anterior margin slightly emarginate, with six setae of which medial four setae equally distributed in medial half, distance from outermost medial seta to lateral seta about twice distance between medial setae. Antenna overall length moderately long, antennomere 11 just reaching beyond pronotal base. *Thorax*. Pronotum transverse, lateral margin sinuate near base, medial and basal setae touching lateral marginal bead; basal impressions obsolete; anterior angles scarcely produced; microsculpture not visible at 50× magnification; entire surface covered with minute punctulae. Elytral striae extremely shallow, scarcely impressed or absent; base of elytra not margined; humeri prominent but rounded; parascutellar punctures present at base of striae 1; interval 1 with two punctures at apex; interval 3 with three (right) or six (left) punctures; interval 5 with three (right) or four (left) punctures; interval 7 with one puncture near apex; interval 9 with 20 evenly spaced punctures; elytral microsculpture visible at 50× as irregular isodiametric mesh of microlines; entire surface with micropunctulae. Male protaromeres 1–3 expanded with squamose setae ventrally. Tarsomere 5 on all legs ventral setose. Metatrochanter without setae. Metacoxa with single lateral seta. *Abdomen*. Abdominal ventrites smooth, glabrous except for very shallowly impressed transverse sulcus on last ventrite. Male with two setae on last ventrite. Aedeagus (Fig. 4) with small, dissimilar conchoid parameres; right paramere with large dorsal process (Fig. 4D); left paramere with a minute transverse apophysis (Fig. 4C). Median lobe with apex truncate; ostium dorsal; endophallus with small median sclerite.

#### Etymology.

The specific epithet *finisterrae* refers to the type locality, the Finisterre Range. Additionally, Finisterre is a contraction that derives from the Latin *Finis Terrae*, meaning “End of the Earth”, an appropriate metaphor for this remote and entomologically little-known region. Although *terrae* is the genitive form, the contraction is treated as a noun in apposition since the “end of the Earth” is used as the name for the location.

#### Habitat.

Only a single specimen of *Lesticus finisterrae* is known. It was collected in an unbaited pitfall trap placed in upper montane moss forest at an elevation of 3050 meters. The area was near the upper limit of forest growth and adjacent to open grassland (Fig. 2). Under the forest canopy, a fairly dense understory of low vegetation was present (Fig. 1).

#### Identification and systematics.

**“**Trigonotomi” sensu Dubault et al. (2008) includes *Lesticus*, *Trigonotoma*, *Euryaptus* and *Pareuryaptus*. Although the group has not been rigorously tested for monophyly, it is reasonable to maintain its use as a working hypothesis based on similarity. Additionally, the genus *Nesites* Andrewes (cotype examined), which has the same combination of characters as *Trigonotoma*, should be included. It is likely that *Trigonotoma* and *Nesites* will prove to be synonymous. *Lesticus finisterrae* has the following states for characters given by Dubault et al. (2008): antennomere 1 less than combined length of antennomeres 2+3; setae at the anterior margin of the labrum more or less equally distributed; three or more setigerous punctures on interval 3 of the elytral disc. Females of *Lesticus* have four setae (two pairs) on the last ventrite, but the female for *Lesticus finisterrae* is unknown. The parascutellar striae are generally present and long in *Lesticus* species; however in *Lesticus finisterrae* all striae are very shallow or not impressed. There is no indication of the parascutellar striae. *Lesticus finisterrae* deviates from other species of *Lesticus* by the extremely prominent eyes, depressed form and lack of sulci on the abdominal ventrites. Based on general similarity and adjacencies of ranges, it seems probable that *Lesticus finisterrae* is an isolated and derived member of the *Lesticus chloronotus* group.

##### Key to pterostichine-like Harpalinae

Below is a revised key to the genera of “pterostichites” of New Guinea based on the key by Darlington (1962: 499–500). We have excluded *Mecyclothorax* Sharp (Moriomorphini, Liebherr 2011), which was included in Darlington’s key, as it is not a member of the Harpalinae and only distantly related. It is easily distinguished from all the included taxa by the presence of a fixed seta in the mandibular scrobe.

**Table:** 

1	Front tibia fossorial, outer apical angle strongly produced; bodyform parallel-sided; antennae moniliform.	*Morion* Latreille [Morionini]
–	Front tibia with outer apical angle not produced; (other characters variable)	2
2	Small, compact; antennae moniliform; elytron with basal pore (if present) at base of 3rd stria	3
–	Size and form variable; antennae usually not moniliform; elytron with basal pore (if present) near or inside base of 2nd stria	5
3	Elytron without basal pore; anteriolateral prothoracic setae almost on anterior angles	*Brachidius* Chaudoir [Cratocerini]
–	Elytron with basal pore at base of 3rd stria; anteriolateral prothoracic setae about 2/5 prothoracic length behind anterior angles	4
4	Elytra ovoid or elongate-ovoid, apex broadly rounded	*Caelostomus* Macleay [Drimostomatini]
–	Elytra elongate-rectangular, bluntly truncate at the apex	*Stegazopteryx* [Drimostomatini]
5	Antennomere 2 attached to 1 more eccentrically than usual; mentum transverse; metacoxal anterior sulcus sinuate; (small, 4.7–6.8mm, in New Guinea hydrophilic species)	*Abacetus* Dejean [Abacetini]
–	Antennomere 2 attached to 1 less eccentrically; (other characters variable, but never in combination as above)	6
6	Four basal antennomeres glabrous; size very large, length (in New Guinea) about 50mm or more	*Catadromus* Macleay [Pterostichini]
–	Three basal antennomeres glabrous; size much smaller	7
7	Abdominal ventrites 4–6 with transversely impressed sulci or margined at base, at least toward sides	8
–	Abdomen with ventral segments not thus impressed or margined, or only ventrite 6 with a very shallowly impressed sulcus	11
8	Elytron with 10th interval absent or not distinct from margin	*Prosopogmus* Chaudoir [Pterostichini]
–	Elytron with a distinct 10th interval at least posteriorly	9
9	Elytra with 3rd intervals impunctate; proepisterna longitudinally strigate	10
–	Elytra with 3rd intervals with setigerous punctures; parascutellar striae present (except when other striae obsolete); proepisterna not strigate (but often punctate)	*Lesticus* (in part) [Pterostichini]
10	Metepisternum short, flight wing reduced	*Rhytiferonia* Darlington [Pterostichini]
–	Metepisternum elongate, flight wing full (in New Guinea)	*Rhytisternus laevis* (Macleay) [Pterostichini]
11	Small size, broad (prothoracic width/length c. 1.55-1.71), compact (superficially similar to *Brachidius* but with antennae less stout and basal pore of elytron present, at base stria 2)	*Cosmodiscus* Sloane [Abacetini]
–	Size small to large, but never so broad and compact	12
12	Elytra with 3rd interval punctate; parascutellar striae absent or nearly so	13
–	Elytra with 3rd interval impunctate; parascutellar striae variable	17
13	Antennae subgeniculate, first antennomere moderately longer than 2 and 3 together	*Homalonesiota* Maindron [Loxandrini]
–	Antennae not at all geniculate, first antennomere shorter than 2 and 3 together	14
14	Metepisterna scarcely longer than wide	15
–	Metepisterna clearly longer than wide	16
15	Elytra without plica. Mentum with prominent epilobes. Mentum tooth prominent, acuminate-entire	*Haploferonia* Darlington [Loxandrini]
–	Elytra with plica. Mentum transverse, epilobes not prominent. Mentum tooth broad and emarginate	*Lesticus* (in part) [Pterostichini]
16	Prothorax not cordate	*Loxandrus* Leconte [Loxandrini]
–	Prothorax cordate	*Nebrioferonia* Straneo [Loxandrini]
17	Very small (4.0–4.5 mm.); parascutellar stria lacking	*Tiferonia* Darlington [Abacetini]
–	Larger; parascutellar stria present	18
18	Flight wings usually (not always) fully developed; body proportions normal, head not relatively very large	*Platycaelus* Blanchard [Pterostichini]
–	Flight wings atrophied; head very large	*Analoma* Darlington [Pterostichini]

**Figure 5.**
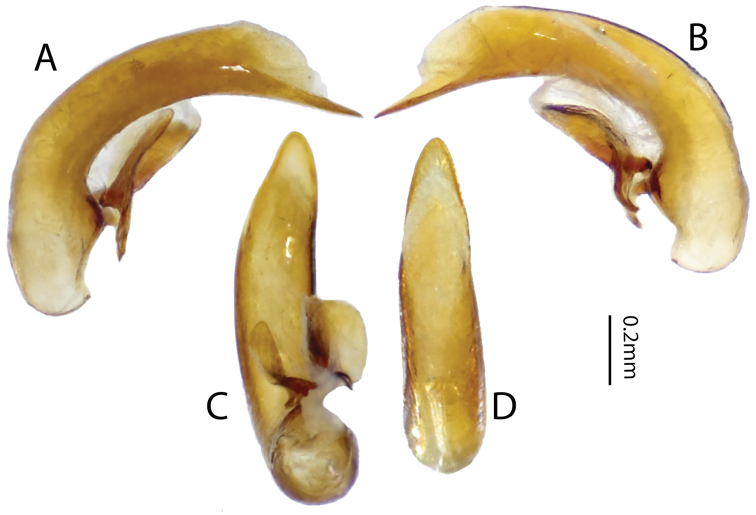
Male genitalia of *Stegazopteryx ivimkaensis*. Median lobe. **A** left lateral **B** right lateral **C** ventral and **D** dorsal views.

##### Additional records

*Stegazopteryx ivimkaensis* Will

A male specimen labeled “INDONESIA W-PAPUA 130km SE Kalmana, Omba (=Yamor) river 10–20km from coast, 4°05'49"S, 134°54'09"E, 10–20m, 09.–11.II.2011 leg. A. Skale (008).” Specimen deposited ZSM.

This specimen record extends the range of this species into eastern West-Papua. This specimen, the two female syntype specimens and the further records below, are all from elevations of 120m and below. This species is now known to range across nearly all of New Guinea. The examined male specimen is consistent with the description: of the genus based on females as given by Will (2004), with the following additions. sbl=5.4mm, greatest width of elytra 2.3mm. Secondary sexual characters- male protarsi not expanded but with spatulate setae ventrally on tarsomeres 1-3. Last ventrite with one pair of setae. Aedeagus right side up in repose. Right paramere larger and conchoid, left paramere small, peg-like. Median lobe of adeagus simple, no evident sclerotized structure on the endophallus (Fig. 5).

Additional locality records (not examined, Martin Baehr, in litt.). PAPUA NEW GUINEA Canopy Mission, Madang Province, Baiteta, Light, Leg. Olivier Missa: 1 male- AR 53, 30-V-1996; 1 male, 1 female- T 2, 31-V-1993; 1 female, X G, 24-IV-1996; 1 female, M 2, 30-IV-1996. One male and two females deposited IRSNB, one male and one female ZSM.

##### *Rhytisternus laevis* (Macleay)

A female specimen labeled “New Guinea, Weam, Aug 1962// H. Olmus collector”. Deposited ANIC. This species is distributed across northern Australia and it has not been previously reported from New Guinea. It is likely a recent accidental introduction or dispersal. We have only seen this single individual from New Guinea and it is unknown if the species is established.

## Supplementary Material

XML Treatment for Lesticus finisterrae
